# Prognostic prediction of lung adenocarcinoma by integrative analysis of RHOH expression and methylation

**DOI:** 10.1111/crj.13574

**Published:** 2023-01-29

**Authors:** Muyu Kuang, Zhenhua Zhou, Zhongyuan Lu, Weina Shen, Haiyan Ge, Xiaoting Tao, Yue Zhao, Lingdun Zhuge, Yihua Sun, Dongmei Ji, Huibiao Zhang

**Affiliations:** ^1^ Phase I Clinical Trial Center Fudan University Shanghai Cancer Center Shanghai China; ^2^ Department of Orthopaedic Oncology, Changzheng Hospital Naval Military Medical University Shanghai China; ^3^ Department of Thoracic Surgery 903th Hospital of PLA Hangzhou China; ^4^ Department of Pulmonary Diseases Huadong Hospital Shanghai China; ^5^ Department of Thoracic Surgery Fudan University Shanghai Cancer Center Shanghai China; ^6^ Department of Head & Neck tumors and Neuroendocrine tumors Fudan University Shanghai Cancer Center Shanghai China; ^7^ Department of Thoracic Surgery Huadong Hospital Shanghai China

**Keywords:** lung adenocarcinoma, methylation, prognosis, RHOH, TCGA

## Abstract

**Background and objective:**

The development of epigenetics holds great promise for diagnosis and treatment of lung adenocarcinoma (LUAD). The purpose of this work was to analyze the correlation between Ras Homolog Gene Family Member H (RHOH) expression and methylation in patients with LUAD and its association with survival.

**Methods:**

Data related to gene expression, DNA methylation, and clinical features of LUAD from The Cancer Genome Atlas (TCGA) database were analyzed. A total of 50 patients were included in verification group. The methylation level of RHOH in verification group was detected by bisulfite amplicon sequencing.

**Results:**

The RHOH methylation level in TCGA cohort was significantly and negatively correlated with its expression level (Cor = −0.5, *p* = 2.687e−33). Patients with hypermethylation and low expression of RHOH had significantly worse prognosis than patients with hypomethylation and low expression of RHOH (TCGA: *p* = 0.004; validation cohort: *p* = 0.006, HR: 4.740, 95% CI: 1.567–14.340).

**Conclusion:**

Our research revealed that RHOH may prove to be a new potential prognostic predictor for LUAD patients.

## Graphical abstract

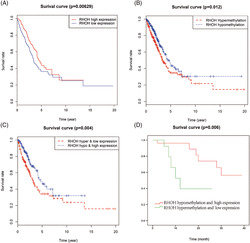

## 1 INTRODUCTION

Lung cancer is one of the most prevalent malignant tumors with high mortality worldwide,^1^ with lung adenocarcinoma (LUAD) as the most common pathological type. In recent years, epigenetic alterations have been described to be closely related to the occurrence and development of LUAD, among which DNA methylation has become the most extensively studied epigenetic form.^2^ Aberrant methylation of genes can occur in the early stage of tumorigenesis, and the degree of aberrant methylation increases with tumor progression. Since the discovery of genome instability caused by genome‐wide hypomethylation, inactivation of tumor suppressor genes due to CpG islands hypermethylation,^3^, ^4^ and inactivation of miRNA due to DNA methylation, DNA methylation has been considered pivotal in LUAD development and progression. Therefore, DNA methylation analysis may provide us with promising targets for the diagnosis and treatment of LUAD.

As a fusion transcript with transcriptional repressor LAZ3/CL6, Ras Homolog Gene Family Member H (RHOH) can negatively modulate IL3 signaling by regulating the JAK‐STAT pathway.^5^ Recent studies have demonstrated that RHOH plays an important role in tumor immune response.^6^, ^7^ Low expression of RHOH was found to be an independent adverse prognostic factor for overall survival (OS) and disease‐free survival in patients with acute myeloid leukemia.^8^ In addition, RHOH has been revealed to regulate the tumor microenvironment.^9^ However, the clinical significance of RHOH in solid tumors has rarely been elucidated, although it was reported as a valuable factor for tumorigenesis.^10^ The aim of the present study was to gain more insights into RHOH and explore the association between RHOH methylation and its expression in LUAD. The results showed that RHOH methylation and gene expression were both related to the prognosis of LUAD patients, and the combined analysis of RHOH methylation and expression could better predict the prognosis of LUAD patients.

## 2 MATERIALS AND METHODS

### 2.1 Cohort

Data related to DNA methylation, mRNA expression, and prognosis of LUAD patients were downloaded from The Cancer Genome Atlas (TCGA) project (https://tcga-data.nci.nih.gov/tcga/). The mRNA expression data included 594 samples (normal: 59 cases and tumor: 535 cases), and the sample size of the methylation profile was 616 cases (normal: 53 cases and tumor: 563 cases). Samples from 50 LUAD patients who underwent surgical resection at our hospital were used as the validation group upon the approval from the ethics committee of the hospital. The final pathological type was determined by postoperative paraffin pathology. Tissues were obtained upon written informed consent from each subject.

### 2.2 Gene ontology (GO) analysis

GO analysis for differentially methylated genes was performed with DAVID 6.8 (https://david.ncifcrf.gov/), knowing that GO can provide a comprehensive resource of computable knowledge about the function of genes and gene products, including the biological process, molecular function, and cellular component. The *p* value < 0.05 was considered statistically significant. GOplot package in R was used to visualize the results.

### 2.3 DNA isolation and methylation analysis

Genomic DNA was extracted using E.Z.N.A.TM Tissue DNA Kit (Omega Bio‐Tek), and its DNA quality and quantity were determined by NanoDrop. Bisulfite amplicon sequencing was performed to detect DNA methylation. A total of 500 ng of genomic DNA were treated with bisulfite using the EZ Methylation‐Gold Kit. In the present study, we used the following Polymerase Chain Reaction (PCR) primers for RHOH methylation sequencing: left primer: TGTTTAATAAAAGTAGGTGAAAATAAAAG and right primer: AAAAAATCATTTAAAACTCTTCTCAATC. After purification of the PCR product, the DNA library was constructed by using the VAHTSTM Turbo DNA library preparation kit for Illumina. All procedures were carried out in accordance with the manufacturers' standard protocol. Deep sequencing was carried out by Illumina Miseq.

### 2.4 Statistical analysis

All TCGA data were standardized before analysis. |Cor| > 0.3 and *p* < 0.05 were considered statistically significant in the correlation analysis of mRNA expression and DNA methylation. Gene expression levels and gene methylation levels were divided into high and low groups by median. Kaplan–Meier survival analysis and Cox regression hazards model were used to estimate the correlation between gene expression, DNA methylation, and the prognosis of LUAD patients. The correlation between RHOH methylation level and clinical characteristics of the LUAD patients was analyzed by chi‐square test (Bonferroni correction was set, and the *p* value for significance was at *p* < 0.006). The correlation between the clinical features and RHOH in TCGA was analyzed by MEXPRESS.^11^ The *p* < 0.05 was considered statistically significant. The correlation between RHOH expression and the prognosis of LUAD patients was analyzed by PREdiction of Clinical Outcomes from Genomic profiles (https://precog.stanford.edu), and the selected Gene Expression Omnibus chips and public database accession numbers were the following: ca00153, ca00182, ca00191, GSE19188, GSE31547, GSE3141, GSE4716, GSE8894, GSE29013, GSE31210, GSE10245, GSE11969, and GSE13213. The software used in this study was Perl, R. The R packages used were "limma", "lush", and "survival".

## 3 RESULTS

### 3.1 Sources of clinical data

A total of 50 LUAD patients were enrolled as the verification cohort of the present study. The clinicopathological data including age, sex, Tumor Node Metastasis (TNM) stage, pathological subtype, and smoking status were collected. The baseline clinical characteristics of the LUAD patients are shown in Table 1.

**TABLE 1:** Correlation between clinicopathological features and RHOH methylation in lung adenocarcinoma

Features	*n*	RHOH hypomethylation	RHOH hypermethylation	χ^2^	*p*
Age					
<61	24	14 (58.3%)	10 (41.7%)	1.974	0.160
≥61	26	10 (38.5%)	16 (61.5%)		
Gender					
Male	22	9 (40.9%)	13 (59.1%)	0.791	0.374
Female	28	15 (53.6%)	13 (46.4%)		
pT					
1	45	24 (53.3%)	21 (46.7%)	5.128	0.077
2	3	0 (0.0%)	3 (100.0%)		
3	2	0 (0.0%)	2 (100.0%)		
4	—	—	—		
pN					
0	46	23 (50.0%)	23 (50.0%)	1.923	0.382
1	2	0 (0.0%)	2 (100.0%)		
2	2	1 (50.0%)	1 (50.0%)		
3	—	—	—	—	—
pM					
0	47	24 (51.1%)	23 (48.9%)	2.946	0.086
1	3	0 (0.0%)	3 (100.0%)		
Stage					
1	44	23 (52.3%)	21 (47.7%)	4.017	0.260
2	2	1 (50.0%)	1 (50.0%)		
3	1	0 (0.0%)	1 (100.0%)		
4	3	0 (0.0%)	3 (100.0%)		
Tumor size					
Small (≤15 mm)	25	16 (64.0%)	9 (36.0%)	5.128	0.024
Large (>15 mm)	25	8 (32.0%)	17 (68.0%)		
Pathology					
Acinar	34	21 (52.9%)	13 (47.1%)	10.319	0.035
Solid	8	3 (50.0%)	5 (50.0%)		
Papillary	4	0 (33.3%)	4 (66.7%)		
Lepidic	3	0 (0.0%)	3 (100.0%)		
Micropapillary	1	0 (0.0%)	1 (100.0%)		
Smoking					
Nonsmoker	38	20 (52.6%)	18 (47.4%)	1.361	0.243
Current or ex‐smoker	12	4 (33.3%)	8 (66.7%)		

*Note*: Median age = 61 years.

Abbreviation: RHOH, Ras Homolog Gene Family Member H.

### 3.2 GO analysis

**FIGURE 1:**
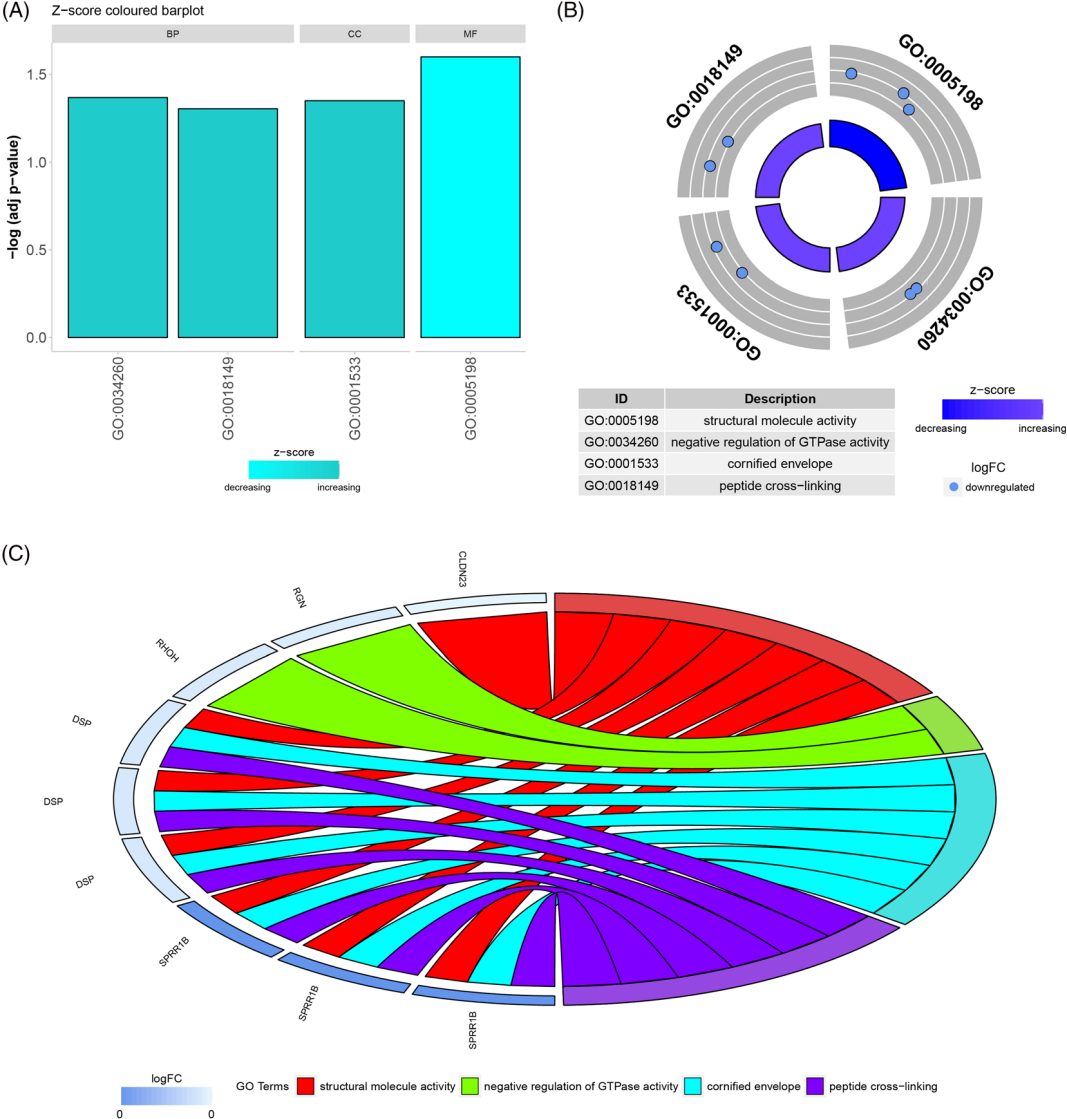
GO analysis of 22 genes related to the prognosis of LUAD patients whose expression level was related to DNA methylation. (A) Structural molecule activity, negative regulation of GTPase activity, cornified envelope, and peptide cross‐linking were found significant different based on those genes by DAVID 6.8. (B) Four GO were downregulated in LUAD. (C) Genes included in the four GO. BP, biological process; CC, cellular component; LUAD, lung adenocarcinoma; MF, molecular function; RHOH, Ras Homolog Gene Family Member H

The expression and methylation of genes related to OS of LUAD patients were analyzed, and the result showed that the expression level of 22 genes was significantly correlated with the methylation status (|Cor| > 0.3 and *p* < 0.05). Further GO analysis of these genes showed four major GOs: structural molecule activity, negative regulation of GTPase activity, cornified envelope, and peptide cross‐linking (Figure 1A). The methylation level of the related genes was relatively low in LUAD (Figure 1B). The molecular function of CLDN23, DSP, and SPRR1B was characterized by structural molecule activity, and the biological processes involved in RGN and RHOH were associated with the negative regulation of GTPase activity (Figure 1C). In the present study, we selected RHOH for further research. The predicted functional partners of RHOH are shown in Figure S1.

### 3.3 RHOH methylation is negatively correlated with RHOH expression

**FIGURE 2:**
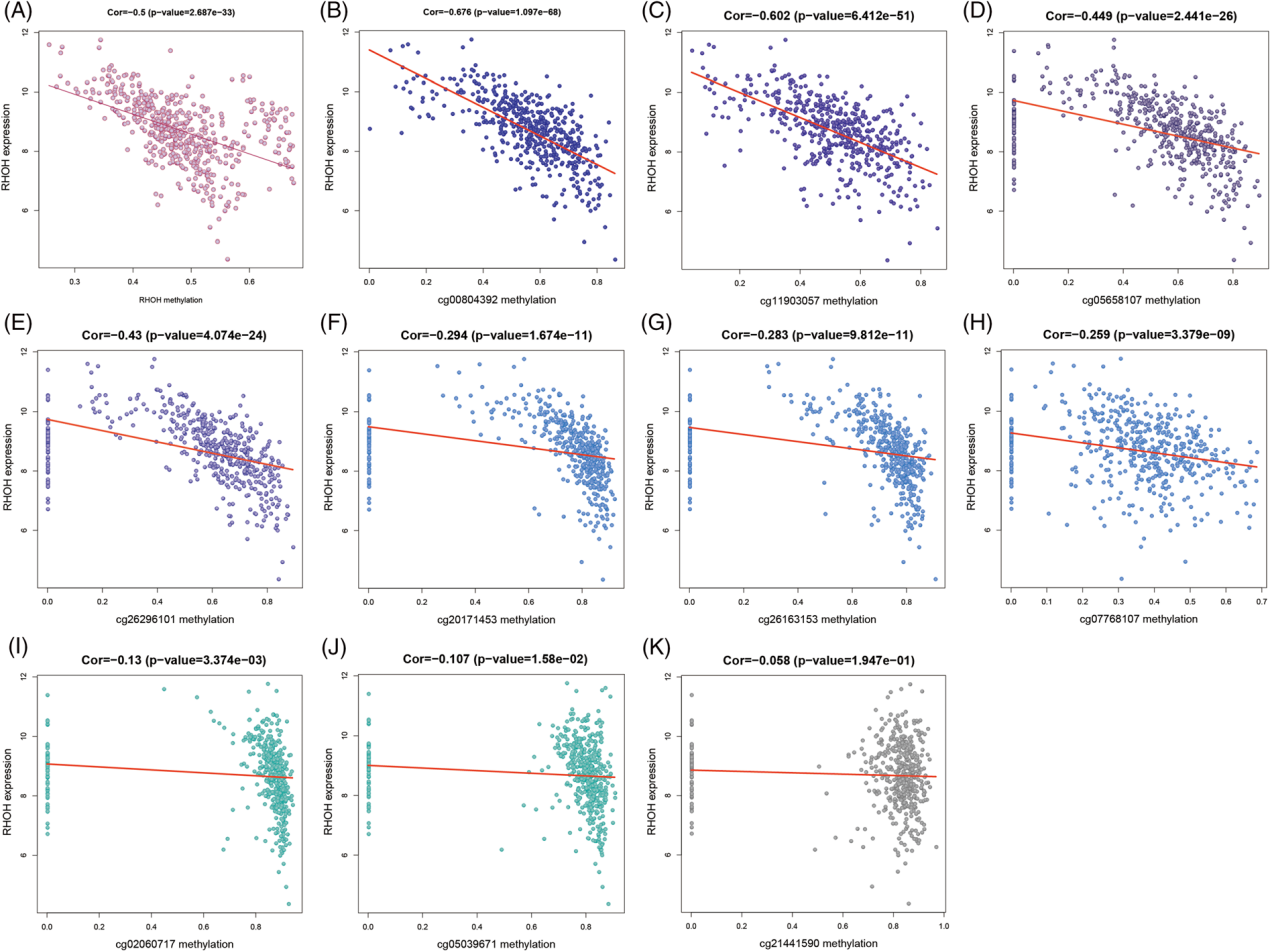
Relationship between Ras Homolog Gene Family Member H (RHOH) expression and DNA methylation. (A) Total. (B–K) Each methylation site

In the current study, we focused on the correlation between DNA methylation and mRNA expression of RHOH and found that the DNA methylation level of RHOH was significantly and negatively correlated with its mRNA expression (Cor = −0.5, *p* value = 2.687e−33) (Figure 2A). TCGA methylation database showed that each methylation site of RHOH was negatively correlated with its gene expression (Figure 2B–K). Among them, four methylation sites were significantly correlated with the expression of RHOH: cg00804392 (Cor = −0.676, *p* value = 1.097e−68), cg11903057 (Cor = −0.602, *p* value = 6.412e−51), cg05658107 (Cor = −0.449, *p* value = 2.441e−26), and cg26296101 (Cor = −0.43, *p* value = 4.074e−24).

### 3.4 Correlation between RHOH methylation, expression, and prognosis

**FIGURE 3:**
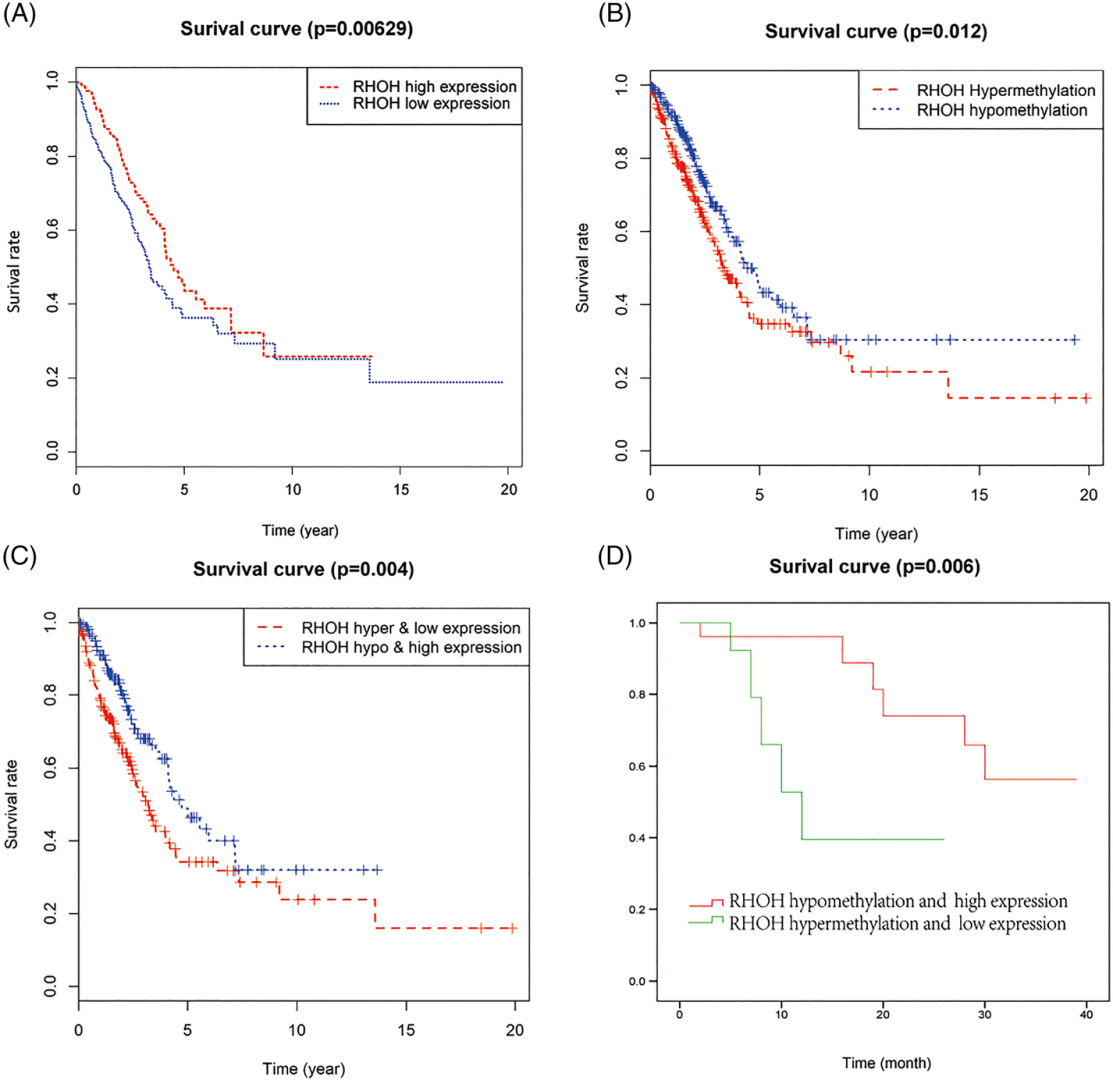
Survival curves of lung adenocarcinoma patients in The Cancer Genome Atlas and validation cohorts. (A) The blue line indicates RHOH high expression patients, and the red line indicates RHOH low expression patients, *p* = 0.00629. (B) The blue line indicates RHOH hypomethylation patients, and the red line indicates RHOH hypermethylation patients, *p* = 0.012. (C) Combined survival analysis of RHOH expression and methylation, *p* = 0.004. (D) Survival curves of lung adenocarcinoma patients in the validation cohort, *p* = 0.006. RHOH, Ras Homolog Gene Family Member H

We identified a significant correlation between RHOH expression and the prognosis of LUAD patients (*p* = 0.00629) (Figure 3A) and found that patients with high RHOH expression had a higher OS rate than those with low RHOH expression. To investigate whether RHOH was associated with OS of LUAD patients, we further collected data of RHOH expression and patient prognosis in PREdiction of Clinical Outcomes from Genomic profiles. It is worth noting that in most datasets, patients with high RHOH expression levels were more likely to survive longer (Figure S2). We next analyzed the association between RHOH methylation and OS in LUAD patients and found that patients with RHOH hypermethylation had a significantly worse prognosis than those with RHOH hypomethylation (*p* = 0.012) (Figure 3B). Integrative analysis of DNA methylation and mRNA expression of RHOH revealed that patients with hypermethylation and low expression of RHOH had a significantly worse prognosis than those with hypomethylation and low expression of RHOH in both TCGA (*p* = 0.004) (Figure 3C) and validation cohort (*p* = 0.006, HR: 4.740, 95% CI: 1.567–14.340) (Figure 3D).

### 3.5 Correlation between RHOH methylation and clinicopathological features

**FIGURE 4:**
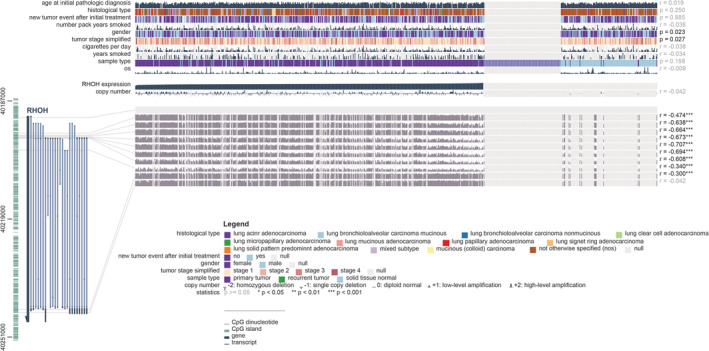
Correlation analysis of RHOH expression and methylation and relationship between RHOH expression and clinical features in The Cancer Genome Atlas cohort by MEXPRESS. RHOH, Ras Homolog Gene Family Member H

The correlation between RHOH expression and the clinical pathologic characteristics of LUAD patients was assessed by MEXPRESS. As shown in Figure 4, the mRNA expression level of RHOH was related to age (*p* = 0.023) and the tumor stage (*p* = 0.027) of LUAD patients. By measuring the methylation of RHOH in LUAD and normal lung tissues from the patients, we investigated the correlation between the clinicopathological features and RHOH methylation level (Table 1).

## 4 DISCUSSION

Studies have revealed the significance of DNA methylation as a promising biomarker for the diagnosis and prognosis of various malignant tumors including lung cancer.^12^, ^13^ DNA methylation and gene expression profiles hold great promise for the diagnosis and treatment of LUAD. Gene methylation alterations of PTEN, RASSF1A, DAPK,^14^ APC,^15^ and TGFBI^16^ were found to be related to the prognosis of LUAD patients. In the present study, we further investigated RHOH by integrative analysis of DNA methylation, mRNA expression, and clinical prognosis of LUAD in TCGA.

Aberrant expression of Rho GTPase has been found in a variety of tumors. The Rho subfamily of GTPase was found to play a vital role in carcinogenesis and tumor progression^17^ and act as a “molecular switch” in many signaling pathways.^18^ High expression of RhoB in testicular cancer and breast cancer was found to be related to tumor progression, but whether it plays a tumor‐promoting or tumor‐suppressing role in colon cancer remains controversial.^19^, ^20^ Overexpression of RhoB in breast cancer was related to tumor progression,^21^ whereas the loss of RhoB expression in advanced lung cancer suggested that it may play a tumor‐suppressing role.^22^ RhoC was involved in the invasion and metastasis of breast cancer, lung cancer, pancreatic cancer, gastric cancer, and other malignant tumors.^23^, ^24^, ^25^, ^26^ RHOH was considered as proto‐oncogene in diffuse large B‐cell lymphoma, suggesting that overexpression of RHOH was associated with poor prognosis.^27^ As RHOH is expressed in hematopoietic cells, it can be used as a liquid biopsy marker for differential diagnosis of tumors.^28^ Abnormal expression of RHOH usually occurs in hematological disease, but it is rarely reported in solid tumors. It was reported that RHOH could promote migration of prostate cancer cells, and the prognosis of prostate cancer patients with high expression of RHOH was relatively poor.^29^ It was found in our study that RHOH was hypermethylated and low‐expressed in LUAD, and the LUAD patients with high expression of RHOH had a favorable prognosis. In addition, the expression of RHOH was related to age and tumor stage; larger‐sample studies are required to verify our findings and conclusions.

RHOH is a membrane‐bound adaptor protein involved in proximal T cell receptor signaling, playing a key role in T cell differentiation. In a study on the screening of markers for early diagnosis of metastatic melanoma, RHOH was identified as central gene involved in immune response and tumor cell progression. In intestinal flora infection, RHOH was involved in the process of immunity and metabolism.^30^ But whether RHOH is involved in the immune response and tumorigenesis of LUAD needs to be further studied.

The important role of Rho GTPase in hematological tumors has been confirmed in recent years,^31^, ^32^ but its role in LUAD has not been reported. We revealed the difference between tumor and normal lung tissue in RHOH methylation based on TCGA database and verified it with Asian population samples, indicating that RHOH may be a potential target for lung cancer treatment. This study has potential limitations that should be taken into account when interpreting the results. In view of the relatively small number of patients included in the validation cohort, it is necessary to conduct a larger study and have sufficient statistical capacity to verify the conclusions. The biological function of RHOH methylation in LUAD needs further study.

In summary, our study demonstrated a correlation between RHOH expression and methylation, which could be used as a biomarker for predicting the prognosis of LUAD patients. In addition, the correlation between RHOH expression and methylation in LUAD was correlated with the clinicopathological features of LUAD patients. In our ongoing study, we will further explore this correlation with immune metabolism in LUAD with the expectation that RHOH could become a new target for the treatment of LUAD.

## AUTHOR CONTRIBUTIONS

Muyu Kuang performed data analysis and wrote the manuscript, and Zhenhua Zhou and Zhongyuan Lu carried out DNA extraction of tissues. Weina Shen, Haiyan Ge, and Xiaoting Tao helped to perform bioinformatics analysis. Yue Zhao and Lingdun Zhuge collected samples and information of clinical cases. Yihua Sun, Dongmei Ji, and Huibiao Zhang conceived of the study and participated in its designation and helped to draft the manuscript. All authors read and approved the final manuscript.

## CONFLICT OF INTEREST

The authors have stated that they have no conflicts of interest.

## ETHICS STATEMENT

All procedures performed in studies involving human participants were in accordance with the ethical standards of the Ethics Committee of Huadong Hospital affiliated to Fudan University.

## Supporting information


**Figure S1.** STRING protein interaction analysis of RHOH.

**Figure S2.** Survival curves concerning RHOH expression level of LUAD patients in PRECOG. LUAD, lung adenocarcinoma.

## Data Availability

The data that support the findings of this study are available from the corresponding author upon reasonable request.

## References

[1] Siegel RL , Miller KD , Fuchs HE , Jemal A . Cancer statistics, 2022. CA Cancer J Clin. 2022;72(1):7‐33. doi:10.3322/caac.21708 35020204

[2] Rizzi G , Lee JR , Dahl C , et al. Simultaneous profiling of DNA mutation and methylation by melting analysis using magnetoresistive biosensor array. ACS Nano. 2017;11(9):8864‐8870. doi:10.1021/acsnano.7b03053 28832112 PMC5810360

[3] Wang P , Qiu W , Dudgeon C , et al. PUMA is directly activated by NF‐κB and contributes to TNF‐α‐induced apoptosis. Cell Death Differ. 2009;16(9):1192‐1202. doi:10.1038/cdd.2009.51 19444283 PMC2872087

[4] Sakai T , Toguchida J , Ohtani N , Yandell DW , Rapaport JM , Dryja TP . Allele‐specific hypermethylation of the retinoblastoma tumor‐suppressor gene. Am J Hum Genet. 1991;48(5):880‐888. 1673287 PMC1683063

[5] Gündogdu MS , Liu H , Metzdorf D , et al. The haematopoietic GTPase RhoH modulates IL3 signalling through regulation of STAT activity and IL3 receptor expression. Mol Cancer. 2010;9(1):225. doi:10.1186/1476-4598-9-225 20738848 PMC2936343

[6] Muro R , Nitta T , Nakano K , Okamura T , Takayanagi H , Suzuki H . γδTCR recruits the Syk/PI3K axis to drive proinflammatory differentiation program. J Clin Invest. 2018;128(1):415‐426. doi:10.1172/jci95837 29202478 PMC5749532

[7] Pan YR , Chen CC , Chan YT , et al. STAT3‐coordinated migration facilitates the dissemination of diffuse large B‐cell lymphomas. Nat Commun. 2018;9(1):3696. doi:10.1038/s41467-018-06134-z 30209389 PMC6135800

[8] Troeger A , Johnson AJ , Wood J , et al. RhoH is critical for cell‐microenvironment interactions in chronic lymphocytic leukemia in mice and humans. Blood. 2012;119(20):4708‐4718. doi:10.1182/blood-2011-12-395939 22474251 PMC3367874

[9] Iwasaki T , Katsumi A , Kiyoi H , et al. Prognostic implication and biological roles of RhoH in acute myeloid leukaemia. Eur J Haematol. 2008;81(6):454‐460. doi:10.1111/j.1600-0609.2008.01132.x 18691253

[10] Wang LX , Li Y , Chen GZ . Network‐based co‐expression analysis for exploring the potential diagnostic biomarkers of metastatic melanoma. PLoS ONE. 2018;13(1):e0190447. doi:10.1371/journal.pone.0190447 29377892 PMC5788335

[11] Koch A , De Meyer T , Jeschke J , Van Criekinge W . MEXPRESS: visualizing expression, DNA methylation and clinical TCGA data. BMC Genomics. 2015;16(1):636. doi:10.1186/s12864-015-1847-z 26306699 PMC4549898

[12] Baglietto L , Ponzi E , Haycock P , et al. DNA methylation changes measured in pre‐diagnostic peripheral blood samples are associated with smoking and lung cancer risk. Int J Cancer. 2017;140(1):50‐61. doi:10.1002/ijc.30431 27632354 PMC5731426

[13] Hao X , Luo H , Krawczyk M , et al. DNA methylation markers for diagnosis and prognosis of common cancers. Proc Natl Acad Sci U S A. 2017;114(28):7414‐7419. doi:10.1073/pnas.1703577114 28652331 PMC5514741

[14] Buckingham L , Penfield Faber L , Kim A , et al. PTEN, RASSF1 and DAPK site‐specific hypermethylation and outcome in surgically treated stage I and II nonsmall cell lung cancer patients. Int J Cancer. 2010;126(7):1630‐1639. doi:10.1002/ijc.24896 19795445

[15] Feng H , Zhang Z , Qing X , Wang X , Liang C , Liu D . Promoter methylation of APC and RAR‐beta genes as prognostic markers in non‐small cell lung cancer (NSCLC). Exp Mol Pathol. 2016;100(1):109‐113. doi:10.1016/j.yexmp.2015.12.005 26681652

[16] Seok Y , Lee WK , Park JY , Kim DS . TGFBI promoter methylation is associated with poor prognosis in lung adenocarcinoma patients. Mol Cells. 2019;42(2):161‐165. doi:10.14348/molcells.2018.0322 30726660 PMC6399005

[17] Haga RB , Ridley AJ . Rho GTPases: regulation and roles in cancer cell biology. Small GTPases. 2016;7(4):207‐221. doi:10.1080/21541248.2016.1232583 27628050 PMC5129894

[18] Mitin N , Rossman KL , Der CJ . Signaling interplay in Ras superfamily function. Curr Biol. 2005;15(14):R563‐R574. doi:10.1016/j.cub.2005.07.010 16051167

[19] Dopeso H , Rodrigues P , Bilic J , et al. Mechanisms of inactivation of the tumour suppressor gene RHOA in colorectal cancer. Br J Cancer. 2018;118(1):106‐116. doi:10.1038/bjc.2017.420 29206819 PMC5765235

[20] Jeong D , Park S , Kim H , et al. RhoA is associated with invasion and poor prognosis in colorectal cancer. Int J Oncol. 2016;48(2):714‐722. doi:10.3892/ijo.2015.3281 26648547

[21] Fritz G , Brachetti C , Bahlmann F , Schmidt M , Kaina B . Rho GTPases in human breast tumours: expression and mutation analyses and correlation with clinical parameters. Br J Cancer. 2002;87(6):635‐644. doi:10.1038/sj.bjc.6600510 12237774 PMC2364248

[22] Mazieres J , Antonia T , Daste G , et al. Loss of RhoB expression in human lung cancer progression. Clin Cancer Res. 2004;10(8):2742‐2750. doi:10.1158/1078-0432.ccr-03-0149 15102679

[23] Griner EM , Dancik GM , Costello JC , et al. RhoC is an unexpected target of RhoGDI2 in prevention of lung colonization of bladder cancer. Mol Cancer Res. 2015;13(3):483‐492. doi:10.1158/1541-7786.mcr-14-0420 25516960 PMC4369172

[24] Wynn ML , Yates JA , Evans CR . RhoC GTPase is a potent regulator of glutamine metabolism and N‐acetylaspartate production in inflammatory breast cancer cells. J Biol Chem. 2016;291(26):13715‐13729. doi:10.1074/jbc.M115.703959 27129239 PMC4919454

[25] Lin M , DiVito MM , Merajver SD , Boyanapalli M , van Golen KL . Regulation of pancreatic cancer cell migration and invasion by RhoC GTPase and caveolin‐1. Mol Cancer. 2005;4(1):21. doi:10.1186/1476-4598-4-21 PMC1173138 15969750

[26] Wu Y , Chen YC , Sang JR , Xu WR . RhoC protein stimulates migration of gastric cancer cells through interaction with scaffold protein IQGAP1. Mol Med Rep. 2011;4(4):697‐703. doi:10.3892/mmr.2011.482 21537845

[27] Duquette ML , Huber MD , Maizels N . G‐rich proto‐oncogenes are targeted for genomic instability in B‐cell lymphomas. Cancer Res. 2007;67(6):2586‐2594. doi:10.1158/0008-5472.can-06-2419 17363577

[28] Zhang YH , Huang T , Chen L , et al. Identifying and analyzing different cancer subtypes using RNA‐seq data of blood platelets. Oncotarget. 2017;8(50):87494‐87511. doi:10.18632/oncotarget.20903 PMC5675649 29152097

[29] Tajadura‐Ortega V , Garg R , Allen R , et al. An RNAi screen of Rho signalling networks identifies RhoH as a regulator of Rac1 in prostate cancer cell migration. BMC Biol. 2018;16(1):29. doi:10.1186/s12915-018-0489-4 PMC5840776 29510700

[30] Sun Y , Zhuang Z , Wang X , Huang H , Fu Q , Yan Q . Dual RNA‐seq reveals the effect of the flgM gene of Pseudomonas plecoglossicida on the immune response of Epinephelus coioides. Fish Shellfish Immunol. 2019;87:515‐523. doi:10.1016/j.fsi.2019.01.041 30708058

[31] Voena C , Chiarle R . RHO family GTPases in the biology of lymphoma. Cell. 2019;8(7):646. doi:10.3390/cells8070646 PMC6678807 31248017

[32] Que F , Zhang L , Wang T , Xu M , Li W , Zang S . RHOA G17V induces T follicular helper cell specification and involves angioimmunoblastic T‐cell lymphoma via upregulating the expression of PON2 through an NF‐κB‐dependent mechanism. Onco Targets Ther. 2022;11(1):2134536. doi:10.1080/2162402x.2022.2134536 PMC9559328 36249275

