# Supplementary figures and images for: Prognostic prediction of lung adenocarcinoma by integrative analysis of RHOH expression and methylation
Source: Clin Respir J. 2023 Jan 29;17(3):148–56. doi: 10.1111/crj.13574 (PMC9978903; doi:10.1111/crj.13574)

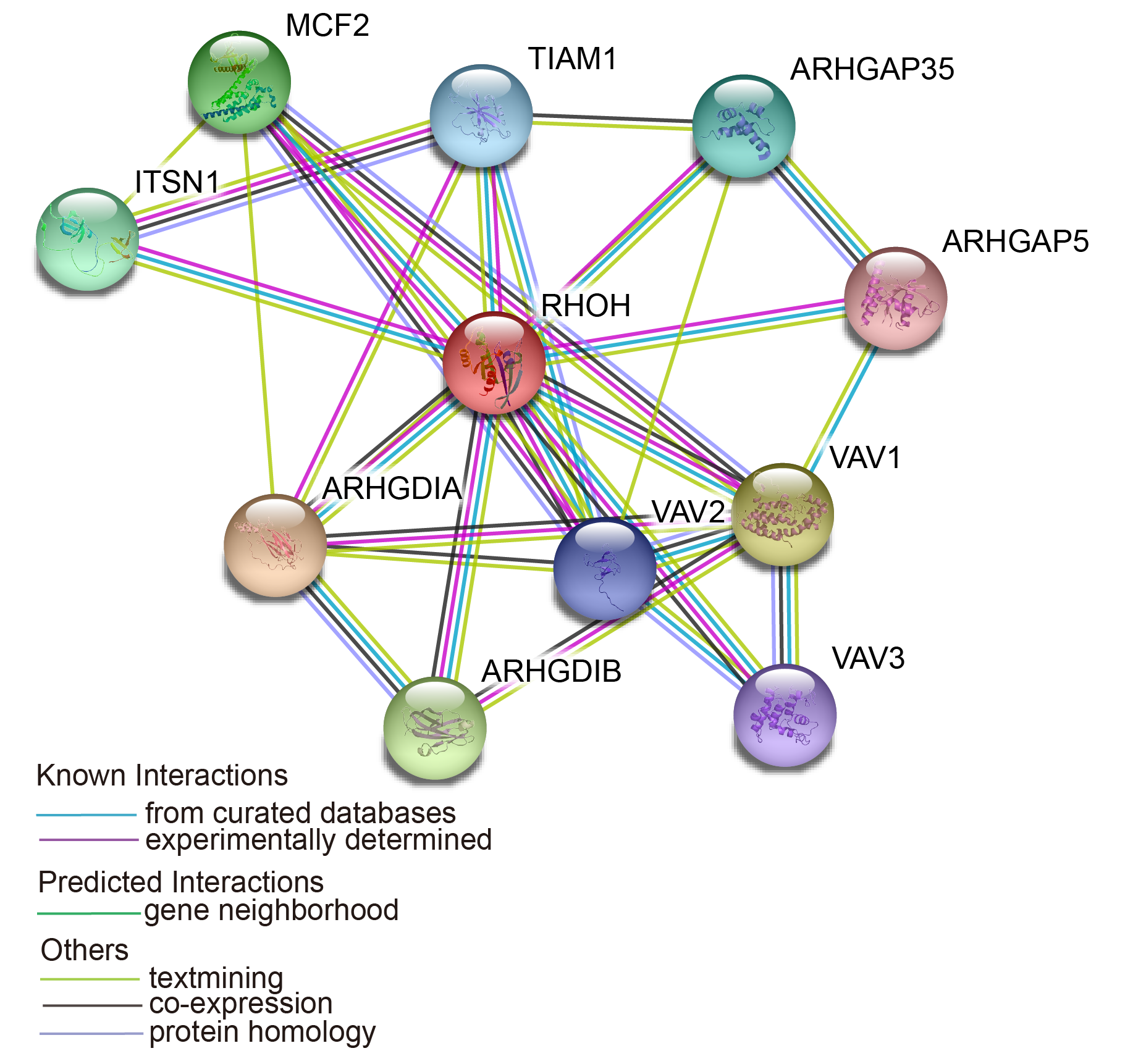

Supplement: Supplementary file 1 — Figure S1. STRING protein interaction analysis of RHOH. [file CRJ-17-148-s002.tif]

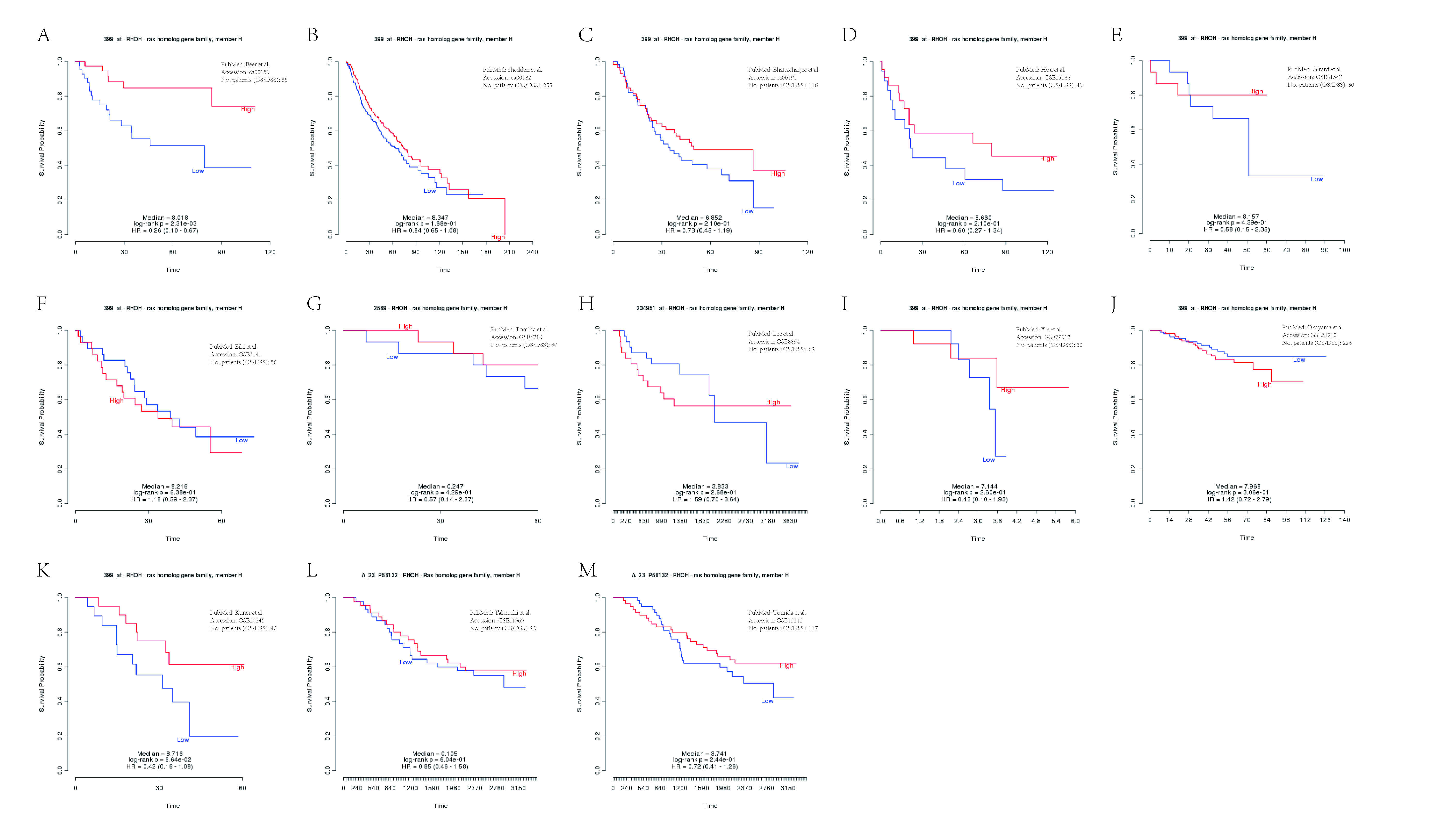

Supplement: Supplementary file 2 — Figure S2. Survival curves concerning RHOH expression level of LUAD patients in PRECOG. LUAD, lung adenocarcinoma. [file CRJ-17-148-s001.tif]
